# Bioinformatics Analysis of Gene Expression Profiles of Sex Differences in Ischemic Stroke

**DOI:** 10.1155/2019/2478453

**Published:** 2019-04-30

**Authors:** Wenhao Zhu, Yinan Nan, Shaoqing Wang, Wei Liu

**Affiliations:** ^1^Department of Encephalopathy, Zibo Hospital of Traditional Chinese Medicine, Zibo, Shandong 255300, China; ^2^International Department, China-Japan Friendship Hospital, Beijing 100029, China; ^3^Department of Traditional Chinese Medicine, Beijing Tiantan Hospital, Capital Medical University, Beijing 100050, China; ^4^Department of Encephalopathy, The Second Affiliated Hospital of Shandong University of Traditional Chinese Medicine, Jinan, Shandong 250001, China

## Abstract

Ischemic stroke (IS) is a complex disease with sex differences in epidemiology, presentations, and outcomes. However, the sex-specific mechanism underlying IS remains unclear. The purpose of this study was to identify key genes contributing to biological differences between sexes. First, we downloaded the gene expression data of GSE22255 from Gene Expression Omnibus (GEO). Differentially expressed genes (DEGs) were identified using R software and related packages. Second, DEGs were separately analyzed by Gene Ontology enrichment and pathways analyses. Third, protein-protein interaction (PPI) network was constructed to further investigate the interactions of DEGs. A total of 123 DEGs were identified between sexes, including 8 upregulated and 115 downregulated genes. In the PPI network, ten key genes were identified, including IL1*α*, IL1*β*, IL6, IL8, CXCL1, CXCL2, CXCL20, CCL4, ICAM1, and PTGS2. Functional enrichment analysis revealed that these genes were mainly enriched in biological processes of immune response and apoptotic process, also in pathways of TNF and NOD-like receptor signaling. In conclusion, the above ten genes may have a protective effect on IS females through their direct or indirect involvement in biological processes of immune response and apoptotic process, as well as in TNF and NOD-like receptor signaling pathways. The results of this study may help to gain new insights into the sex-specific mechanisms underlying IS females and may suggest potential therapeutic targets for disease treatment.

## 1. Introduction

Worldwide, stroke is ranked as the second most common cause of death behind ischemic heart disease, with about 17 million new cases and 6 million deaths each year [1]. It is the third and fifth leading cause of death in women and men in the United States, respectively [2]. Multiple evidences have shown an increase in the prevalence of stroke in recent decades, particularly in developing countries. By 2030, it is expected that an additional 3.4 million Americans will have a stroke, an increase of 20.5% from 2012 estimates [2]. Stroke is characterized by signs and symptoms of numbness or weakness of one side of body, confusion or trouble speaking or trouble understanding others, or dizziness. It significantly decreases the life quality of victims and creates huge public health burden. Generally, strokes can be classified into two major types, namely, ischemic stroke (IS) and hemorrhagic stroke. IS is currently the predominant type of stroke, accounting for almost 87% of stroke cases [2]. Approximately, half of stroke deaths result from IS [3].

Recently, accumulating evidences highlight sex differences in the IS incidence. The incidence of IS is higher among men than among women in most age groups. The prognosis of IS has also been reported differed between sexes. For instance, Rutten-Jacobs et al. [4] found that 20-year mortality after IS was higher in male than in female survivors. Moreover, the symptoms between IS males and females have also been found to differ as well. Women often experience nontraditional stroke symptoms, such as altered mental status, paralysis, decreased level of consciousness, and a generalized numbness or weakness, while men more often experience sensory loss, dysarthria, diplopia, ataxia, and walking problems [5]. The US National Institute of Health has recognized that understanding the biological differences between sexes is imperative to development of effective therapies [6]. Great efforts have been exerted to elucidate the pathophysiology of IS; yet it is still unclear how sex may modify these differences in IS.

Microarrays based on high-throughput platforms for the profiling of genome-wide expression emerge as a promising and efficient tool to identify genomic variants that modulate the risk to develop IS. To identify key genes contributing to biological differences between sexes, we conducted a comprehensive bioinformatics analysis based on gene expression microarray dataset. This study may provide new insights into the sex-specific mechanism underlying IS and may suggest potential therapeutic targets for disease treatment.

## 2. Materials and Methods

### 2.1. Microarray Data

The gene expression data for the present study was obtained from Gene Expression Omnibus (GEO) database (https://www.ncbi.nlm.nih.gov/geo/) using the accession number GSE22255 [7]. The dataset was based on the platform of the GPL570 [HG-U133_Plus_2] Affymetrix Human Genome U133 Plus 2.0 Array (Affymetrix, Santa Clara, California, USA). A total of 40 serum samples were included in the dataset, including 10 serum samples from IS males, 10 from IS females, and 20 from healthy controls. According to the aim of this study, 20 IS samples were selected for further analysis. All of IS patients were adult Caucasians with a mean age of 60.2±10.6 years. All of them were also required to have suffered only one stroke episode at least six months before blood collection.

### 2.2. Differential Expression Analysis

R software and related R packages were used to normalize and analyze differentially expressed genes (DEGs). Firstly, the dataset was normalized by log_2_ transformation in R software. Then, DEGs between IS females and males were screened by Linear Models for Microarray Data (limma) package in R. Significant genes were selected with thresholds of |log_2_⁡ fold  change  (FC)| ≥ 1.58 and adjusted* p* value < 0.05.

### 2.3. Functional Enrichment Analysis

To further analyze biological processes of DEGs in IS females compared with IS males, functional enrichment analysis for DEGs was carried out through the Database for Annotation, Visualization and Integrated Discovery (DAVID version 6.8, https://david.ncifcrf.gov/) [8]. Gene Ontology (GO) terms and the Kyoto Encyclopedia of Genes and Genomes (KEGG) pathways were regarded as enriched with thresholds of* p* value <0.05 and an enriched gene count >2.

### 2.4. PPI Network Analysis

The Search Tool for the Retrieval of Interacting Genes (STRING, version 10.5, https://www.string-db.org/) [9] is a well-known online database and web tool to predict the interactions among the products of DEGs. In this study, the PPI network was constructed via STRING with the default threshold of a combined score > 0.4, and then the PPI network was visualized by Cytoscape (version 3.6.1) [10]. In addition, nodes represent biological molecules and edges connect the nodes to indicate their relationship [11]. The pivotal nodes in the PPI network were identified based on their connectivity degrees.

## 3. Results

### 3.1. Identification of DEGs

**Figure 1:**
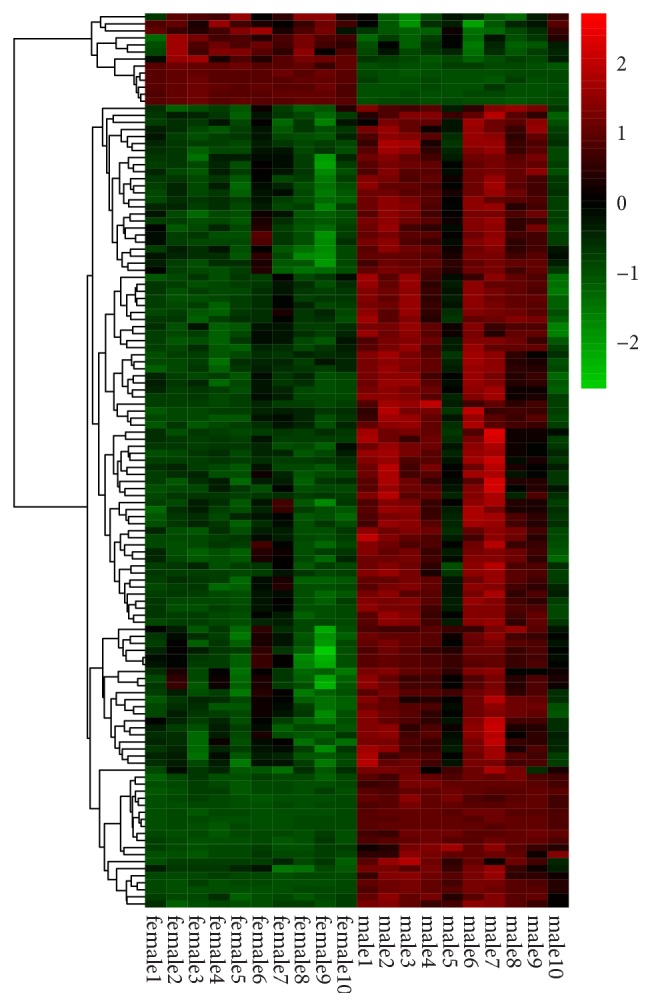
Gene expression value of ischemic stroke (IS) women and men.

According to the cut-off criteria (|log_2_⁡FC| ≥ 1.58 and adjusted* p* value < 0.05), a total of 123 DEGs were identified in serum samples from IS females versus IS males, including 8 upregulated and 115 downregulated genes. The list of DEGs was visualized using hierarchical clustering to generate a heatmap, which could clearly distinguish IS female samples from IS male samples (Figure 1). The top 8 upregulated and 10 downregulated genes were shown in Table 1.

### 3.2. GO Enrichment Analysis

**Figure 2:**
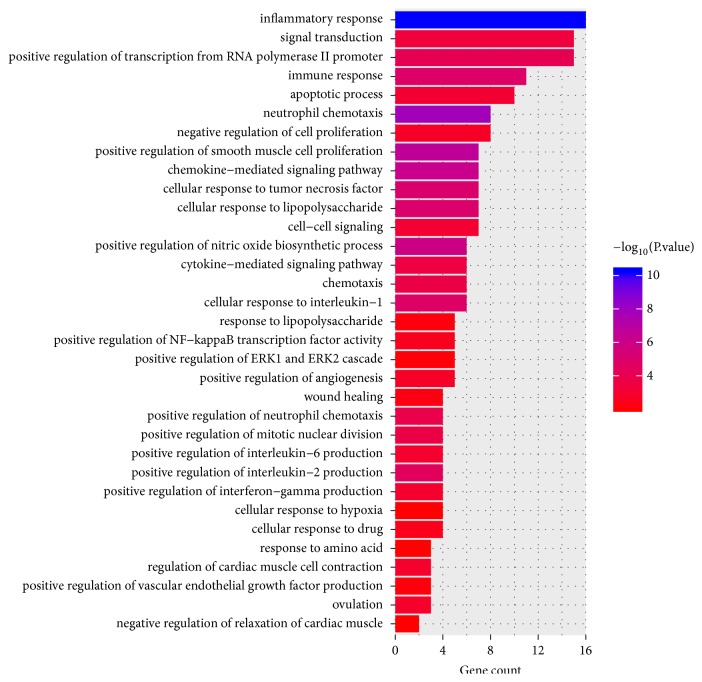
Gene Ontology (GO) enrichment analysis of differentially expressed genes (DEGs) of ischemic stroke (IS) women and men.

GO enrichment analysis was performed to gain a further insight into the biological processes of the selected DEGs related to IS females. As shown in Figure 2, total 33 GO terms were significantly enriched for DEGs, mainly including inflammatory response (*p*=5.47E-11, which involved CXCL1, CXCL2, IL1*α*, IL6, CCL4, and PTGS2), signal transduction (*p*=3.58E-04, which involved CXCL1 and IL1*β*), positive regulation of smooth muscle cell proliferation (*p*=2.10E-07, which involved IL6 and PTGS2), immune response (*p*=1.18E-05, which involved CXCL1, CXCL2, CXCL20, IL1*α*, IL1*β*, IL6, and CCL4), and apoptotic process (*p*=6.85E-04, which mainly involved IL6 and IL1*β*).

### 3.3. KEGG Pathways Analysis

**Figure 3:**
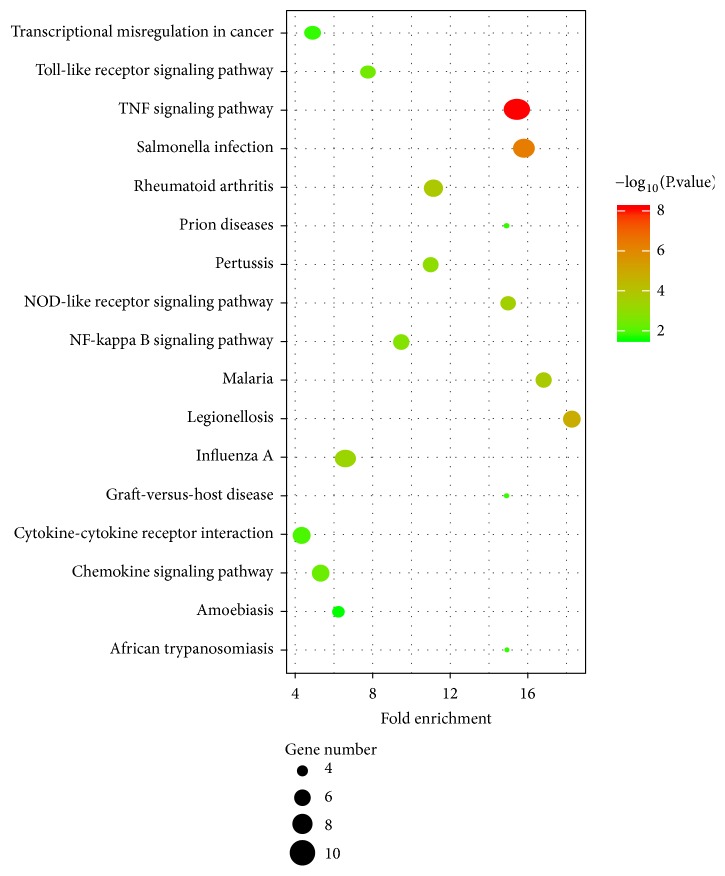
Enriched pathways of differentially expressed genes (DEGs) in ischemic stroke (IS) women and men analyzed by the Kyoto Encyclopedia of Genes and Genomes (KEGG).

KEGG pathways analysis was used to gain a deeper insight into pathways of the screened DEGs in our study. As shown in Figure 3, a total of 17 pathways were enriched mainly in pathways of TNF signaling pathway (*p*=7.77E-09, which involved CXCL1, CXCL2, CXCL20, IL1*β*, IL6, ICAM1, and PTGS2), NOD-like receptor signaling pathway (*p*=2.92E-04, which involved IL1*β* and IL6), and chemokine signaling pathway (*p*=4.57E-03, which involved CXCL1, CXCL2, CXCL20, and CCL4).

### 3.4. PPI Network Construction

**Figure 4:**
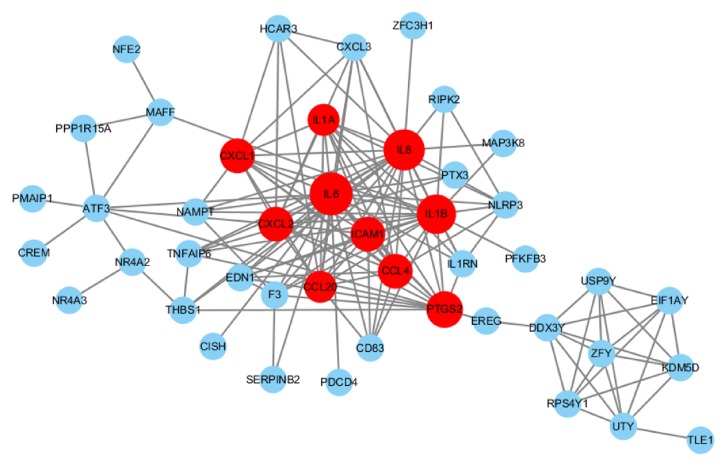
Protein-protein interaction (PPI) network associated with sex differences in ischemic stroke (IS). The red ones represent hub genes with node degree greater than or equal to 10.

In order to better understand the interactions of DEGs, PPI network construction was conducted using the STRING database. As shown in Figure 4, the hub genes with node degree greater than or equal to 10 were IL1*α*, IL1*β*, IL6, IL8, CXCL1, CXCL2, CXCL20, ICAM1, CCL4, and PTGS2. Interestingly, all of hub genes were downregulated in the serum samples from IS females. Among these genes, IL6, IL8, and IL1*β* demonstrated the highest node degrees, which were 23, 22, and 21, respectively.

## 4. Discussion

IS is a complex neurological disorder with substantial morbidity and mortality. It is characterized with sex differences in terms of etiology, risk factors, and outcomes. Sex hormones (oestrogen and androgen), sex chromosomes (XX compared with XY), and social and environmental factors all help explaining these sex differences, albeit partly. The past decade witnessed substantial breakthroughs in the genetics of many types of diseases, including IS. A large number of genetic analyses of IS between sexes has been performed in animal models. Unfortunately, few microarray analyses have been attempted in sex differences of human IS. Tian et al. [12] first examined the effects of sex on RNA expression using whole-genome microarrays by the comparison of human blood from IS cases with healthy controls. Recently, several studies identified key genes between IS cases and controls in human blood [7, 13–15]. However, the exact mechanism underlying sex differences in IS remains poorly understood.

In our study, we aimed to determine sex differences of gene expression in the human serums of IS females compared with IS males using whole-genome microarrays. A total of 123 DEGs were identified between sexes, including 8 upregulated and 115 downregulated genes. In the PPI network, ten key genes were identified, including IL1*α*, IL1*β*, IL6, IL8, CXCL1, CXCL2, CXCL20, CCL4, ICAM1, and PTGS2. Interestingly, all these genes were downregulated in IS women and upregulated in IS men. IL1*α*, IL1*β*, IL6, and IL8 are all members of the interleukin cytokine family. Both IL1*α* and IL1*β* belong to the IL1 cytokine family and mediate inflammation process and involve in various immune responses, inflammatory processes [16]. A recent study reported that IL1, IL6, and TNF-*α* in serum and gingival crevicular fluid were higher in IS patients than healthy controls [17]. Based on our network construction results, IL6 and IL8 seem to be more important than other genes. IL6 is a pleiotropic cytokine which plays a crucial role in the acute inflammatory response [18]. Previous studies showed that the elevated levels of serum and cerebrospinal fluid IL6 were associated with poor stroke prognosis [19]. Similarly, a recent study reported the positive correlation between the higher serum IL8 level and severe disability after IS [20]. CXCL1 and CXCL2 are the main chemokines responsible for neutrophil extravasation. CXCL1, also known as growth-related oncogene, was reported to be elevated in the cerebrospinal fluid of stroke patients during the immediate early phases [21]. PTGS2, also known as Cyclooxygenase 2 (COX 2), is a crucial enzyme in prostaglandin biosynthesis [22]. A meta-analysis with 4,086 IS cases and 4,747 controls suggested that the variant of G-765C allele of PTGS2 may contribute to the IS incidence, specifically in Brazilians and the African-Americans [23]. Taken together, one possible explanation of higher incidence and worse outcomes of IS men is the role of these upregulated key genes.

Altered gene expression affects proteins and pathways in numerous biological functions in IS. We found that these genes were mainly enriched in biological processes of immune response and apoptotic process, as well as in pathways of TNF and NOD-like receptor signaling. An increasing number of studies demonstrated that immune response was implicated in both the manifestation and evolution of brain ischemia [24]. TNF belongs to the tumor necrosis factor family and prominent members include TNF-*α* and TNF-*β*. Previous studies have provided many evidences that TNF-*α* induces the expression of IL1, IL6, and cell necrosis or apoptosis. Higher serum TNF-*α* level was also associated with poor outcomes after stroke [25]. The NOD-like receptors are a family of cytosolic proteins involved in the recognition of intracellular pathogens [26]. The NOD-like receptor protein 3 inflammasome is a multiprotein which is the most frequently studied. It serves as a key mediator of the immune response which contributes to neurovascular unit damage in stroke [27]. Furthermore, interleukin inhibitors, such as IL1, IL6, and TNF-*α* inhibitors, have been recognized as promising therapy in the treatment of immune and inflammatory related diseases. Based on our findings, we strongly suggest that immune therapies could be selectively taken into account in the prevention and treatment of IS. Further biological studies are still warranted to confirm our findings.

In conclusion, the ten above genes we have identified may have a protective effect on IS females through their direct or indirect involvement in biological processes of immune response and apoptotic process, as well as in TNF and NOD-like receptor signaling pathways. The results of this study may help to gain new insights into the sex-specific mechanisms underlying IS and may suggest potential therapeutic targets for disease treatment.

## Figures and Tables

**Table 1:** Top 8 up- and 10 downregulated differentially expressed genes of ischemic stroke in women versus men.

Gene ID	Gene symbol	Adj. *p* value	logFC	Up- or downregulation
224588_at	XIST	9.54E-23	8.47	Up
206207_at	CLC	2.54E-17	5.09	Up
231592_at	TSIX	7.49E-06	2.18	Up
204069_at	MEIS1	2.26 E-03	1.77	Up
228195_at	C2orf88	6.96 E-03	1.68	Up
209930_s_at	NFE2	7.56 E-03	1.62	Up
1559477_s_at	MEIS1	1.40 E-03	1.60	Up
223377_x_at	CISH	2.21 E-03	1.59	Up
201909_at	RPS4Y1	2.76E-23	-7.46	Down
205000_at	DDX3Y	1.45E-19	-7.24	Down
204470_at	CXCL1	4.96E-21	-5.84	Down
207850_at	CXCL4	8.97 E-03	-5.15	Down
39402_at	IL1*β*	7.11E-14	-5.01	Down
210118_s_at	IL1*α*	8.14 E-04	-4.75	Down
205476_at	CCL20	1.536 E-03	-4.64	Down
1569203_at	CXCL2	6.72E-13	-4.53	Down
205207_at	IL6	3.60 E-03	-4.26	Down
228492_at	USP9Y	4.22E-21	-4.08	Down

## Data Availability

The data used to support the findings of this study are available from the corresponding author upon request.
